# Mixed mating system and intraspecific variation in lizard pollination of *Withania frutescens* (L.) Pauquy

**DOI:** 10.1093/aobpla/plaf008

**Published:** 2025-02-15

**Authors:** Raquel Muñoz-Gallego, Anna Traveset, Rafel Beltrán, Sandra Hervías-Parejo

**Affiliations:** Global Change Research Group, Mediterranean Institute for Advanced Studies, IMEDEA, CSIC-UIB, C/ Miquel Marquès, 21, 07190 Esporles, Balearic Islands, Spain; Department of Natural Systems and Resources, Universidad Politécnica de Madrid, Ciudad Universitaria, ES-28040 Madrid, Spain; Global Change Research Group, Mediterranean Institute for Advanced Studies, IMEDEA, CSIC-UIB, C/ Miquel Marquès, 21, 07190 Esporles, Balearic Islands, Spain; Global Change Research Group, Mediterranean Institute for Advanced Studies, IMEDEA, CSIC-UIB, C/ Miquel Marquès, 21, 07190 Esporles, Balearic Islands, Spain; Instituto Politécnico Nacional, CIIDIR, Unidad Oaxaca, C. Hornos 1003, Santa Cruz Xoxocotlán, 71233 Santa Cruz Xoxocotlán, Oax, Mexico; Global Change Research Group, Mediterranean Institute for Advanced Studies, IMEDEA, CSIC-UIB, C/ Miquel Marquès, 21, 07190 Esporles, Balearic Islands, Spain

**Keywords:** autogamy, cryptic dioecy, double mutualism, functional dioecy, florivory, islands, *Podarcis lilfordi*, reproductive system

## Abstract

Plant reproductive strategies are particularly relevant on islands, where environmental constraints usually shape ecological dynamics. In this sense, the role of lizards (Lacertilia) as flower visitors and potential pollinators has been increasingly recognized. However, lizards may also consume plant reproductive tissues, potentially influenced by lizard intraspecific traits such as age and sex. This study aims to investigate, for the first time, the reproductive biology of the rare Mediterranean shrub *Withania frutescens* (L.) Pauquy (Solanaceae), and to assess the role of the Balearic lizard *Podarcis lilfordi* Günther (Lacertidae) as a potential pollinator on Na Redona islet (Cabrera archipelago, Balearic Islands). We analysed flower traits (corolla length, corolla diameter, stamen length, and pistil length) and performed flower bagging experiments with three pollination treatments (open pollination, autogamy, and cross hand-pollination) from 2018 to 2021 to unravel the plant reproductive system. Fruit set, the number of seeds per fruit, seed weight, size and viability were then assessed. Observational censuses were conducted to identify the main flower visitors and estimate their visitation frequency. Finally, we measured morphometric traits of lizards and explored potential intraspecific variation in floral use. The flowers of *W. frutescens* were morphologically hermaphroditic but functionally dioecious, spatially separated in unisexual individual plants. Open pollination and autogamy treatments resulted in similar fruit set, while cross hand-pollination produced the highest value. However, open pollination significantly increased seed weight and viability. Lizards were the most frequent floral visitors, accounting for 68% of visits, while insects made up the remaining 32%. Lizards played a dual role as both pollinators and florivores, with 45% of their visits potentially contributing to pollination and 55% involving florivory. Juveniles and females primarily conducted legitimate visits, whereas males –with a larger size- were more likely to consume the flowers. Our research describes, for the first time, a mixed reproductive system in *W. frutescens*, combining hermaphroditism with cryptic dioecy. We also provide a new example of a lizard-pollinated plant, highlighting the importance of vertebrates on island pollination as well as the influence of pollinator intraspecific variation on plant reproductive success. Further research on the reproductive and pollination systems of small, isolated plant populations is crucial, given their heightened vulnerability to disturbance and genetic inbreeding.

## Introduction

Reproductive strategies in plants are fundamental to their survival, influencing not only their capacity to reproduce but also shaping interactions within ecosystems and driving evolutionary processes (Holsinger 2000). When pollinators become scarce, self-compatible plant species may be more resilient (i.e. reproductive assurance; e.g. Balao et al. 2024) than species that rely on pollinator visits for reproduction (Shivanna 2015). This adaptive advantage is particularly relevant to island environments, where self-compatible species are more prevalent, supporting successful colonization (Grossenbacher et al. 2017; Razanajatovo et al. 2019). In fact, in a sample of >1500 species from the Asteraceae, Brassicaceae, and Solanaceae families, 66% of island species were self-compatible, in contrast to 41% of mainland species (Grossenbacher et al. 2017). However, while self-compatibility provides resilience, it may also reduce genetic variability, making plants less adaptable to environmental disturbances (Holsinger 2000). Consequently, elucidating both the reproductive systems and pollination that shape the genetic structure of plant populations is essential for identifying reproductive limitations, particularly in conservation and management strategies for insular plants (Tandon et al. 2020).

Among angiosperms, the Solanaceae is a large family comprising about 100 genera and more than 2000 species across all continents except Antarctica. Within the genus *Withania,* which includes 26 species worldwide (Mir et al. 2013), research has mainly focused on medicinal properties (particularly for *W. somnifera*; e.g. Palliyaguru et al. 2016), but the reproductive biology and ecology remain understudied. *Withania* is characterized by hermaphroditic flowers and varied breeding systems, ranging from obligate selfing to obligate out-crossing (e.g. Anderson et al. 2006; Lattoo et al. 2007; Mir et al. 2013). Only three cases of dioecy within the genus have been proposed so far: *W. adpressa* (Hepper 1991), *W. coagulans* (Hunziker 2001), and *W. aristata* (Anderson et al. 2006). Pollination is generally entomophilous (Knapp 2010), although some Solanaceae species are pollinated by birds (e.g. genus *Nicotiana*; Kaczorowski et al. 2005) or bats (e.g. *Dyssochroma viridiflorum*; Sazima et al. 2003).

From a global perspective, the role of vertebrates as flower visitors has gained more attention in recent decades (Ratto et al. 2018 and references therein), particularly on islands where limited resources force vertebrates to expand their dietary niches (Olesen and Valido 2003; Traveset et al. 2015). Both birds and reptiles have been shown to play a crucial role as effective pollinators of insular plants (Hervías-Parejo and Traveset 2018; Romero-Egea et al. 2023; González-Castro and Siverio 2024; Robles et al. 2024). However, intraspecific variation in flower-visiting behaviours, including age and sex, may influence pollination success and plant reproductive outcomes (Rodríguez-Rodríguez et al. 2013; Fuster and Traveset 2019, 2020). For instance, the pollination effectiveness of hummingbirds has been shown to vary with individual foraging behaviour (Maruyama et al. 2016). Similarly, the dietary expansion driven by insular ecological constraints may push vertebrates to consume not only nectar and pollen but also petals and floral reproductive organs. Smaller lizards, such as females and juveniles, can access smaller corollas and act as legitimate pollinators, whereas larger lizards, such as males, often act as florivores (Fuster and Traveset 2020; Romero-Egea et al. 2023). Therefore, a comprehensive understanding of the contribution of vertebrates to plant reproduction requires consideration of such intraspecific variability.

Here, we aimed to assess, for the first time, the reproductive biology of an insular population of the rare Mediterranean shrub *Withania frutescens* (L.) Pauquy, and the role of lizards as its potential pollinators. Our specific objectives were to (i) determine the plant reproductive system; (ii) identify its main pollinators, (iii) evaluate whether there are intraspecific differences (among juveniles, females and males) in flower use by lizards. Previous observations suggest that *W. frutescens* may have two flower morphs, indicating some form of dioecy. Moreover, the Balearic lizard *Podarcis lilfordi* Günther is described to visit flowers of a wide array of Mediterranean plants (e.g. Justicia-Correcher et al. 2023; Romero-Egea et al. 2023) and, particularly, of *W. frutescens* (Hervías-Parejo et al. 2024), in addition to effectively disperse its seeds (Castilla 2000). Given that insect pollinators are generally less abundant on islands compare to the mainland (Olesen and Valido 2003; Traveset et al. 2016), we predict that lizards will play a more relevant role as pollinators of *W. frutescens* than other floral visitors, representing a new case of double mutualism (*sensu*Hansen and Müller 2009). Additionally, we expect intraspecific differences in flower use by lizards, as described for other plant species (Romero-Egea et al. 2023).

## Methods

### Study species

**Figure 1.**
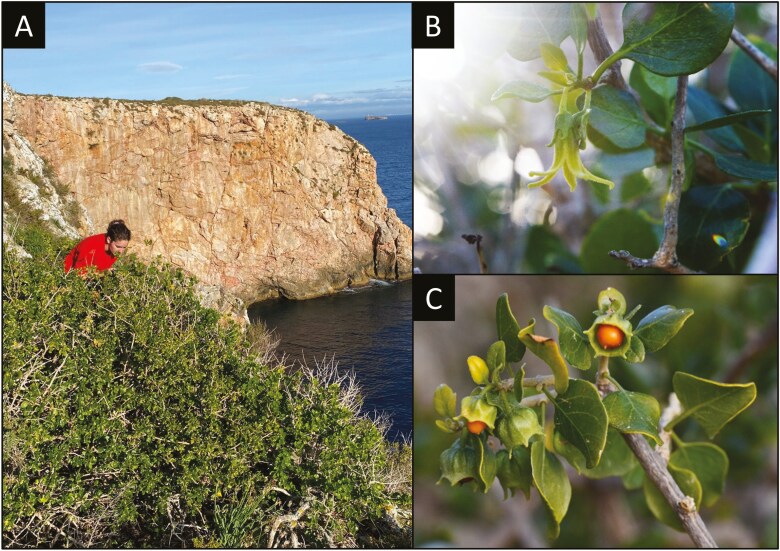
(A) Large individual of *W. frutescens* located in a cliff of Na Redona (Cabrera Archipelago National Park, Balearic Islands). (B) Bell-shaped flower of *W. frutescens*. (C) Ripening fruits of *W. frutescens.*

*Withania frutescens* (L.) Pauquy is a relatively rare plant practically restricted to the West-Mediterranean region, found in coastal habitats of southeastern Iberian Peninsula (Andalucía, Región de Murcia and Comunidad Valenciana), the Balearic Islands, Morocco, Algeria, and the Canary Islands. This shrub usually reaches 1 to 3 meters in height and up to 5 meters in width (Fig. 1A). It experiences several flowering episodes throughout the year, especially in autumn and spring. The flowers have five petals and are pale greenish-yellow with a typical bell (Fig. 1B). The plant produces green fruits that turn yellow to red when ripe (Fig. 1C). It is considered a salt- and drought-tolerant species, but plant orientation and climatic conditions seem to influence its growth (Bouissane and Bailly 2024).

### Study area

Fieldwork was conducted from 2018 to 2021 on Na Redona (39°10′5″N, 2°58′35 ″E), an islet approximately 11 ha in size and 56 m high, located in the Cabrera Archipelago National Park (Balearic Islands, Western Mediterranean Sea). The islet’s habitat is typical Mediterranean shrubland, dominated by species such as *Suaeda vera*, *Olea europaea, Lavatera maritima, Euphorbia dendroides* and *Withania frutescens*. The climate is characterized by dry, hot summers and mild winters with little rainfall. The average annual temperature and cumulative precipitation were 18.2°C and 493 mm in 2018, 18.1°C and 318.8 mm in 2019, 18°C and 424.4 mm in 2020, and 17.6°C and 405.2 mm in 2021, respectively (data from the Balearic weather station network *Balears Meteo;*http://balearsmeteo.com/). The islet lacks carnivores, resulting in a high density of the Balearic lizard *P. lilfordi* (around 1000 lizards·ha^−1^; Pérez-Cembranos and Pérez-Mellado, 2024). Moreover, it is the only terrestrial vertebrate on the islet, apart from birds, as introduced rats (*Rattus rattus*) were eradicated in 2008 (M. McMinn, *pers. comm.*).

### Flower traits and plant reproductive system

From 2018 to 2021, flower traits such as corolla length, corolla diameter at opening, stamen length, and pistil length were measured, using a digital calliper, in 10–15 flowers in a total of 19 reproductive individuals of *W. frutescens*. Plant height, plant width, and the total number of available flowers were also recorded for each individual plant. From 2019 to 2021, 23 reproductive individuals in total were selected for reproductive experiments, whereas 11.1 ± 0.9 (mean ± SE) flowers per plant where randomly assigned to three pollination treatments: (i) open pollination, i.e. flowers were left open to natural pollination; (ii) autogamy, i.e. flowers were bagged before anthesis; (ii) cross hand-pollination, i.e. pollen was taken from functionally male flowers of another plant and applied to the stigmas of the selected flowers. When the fruits were ripe, the bags were removed, and the fruits from each treatment were collected and counted to estimate fruit set (= number of fruits/number of flowers) and the number of seeds produced per fruit. Seed weight and seed width (*n* = 156) were then measured using a precision balance (to the nearest 0.1 mg) and a digital calliper, respectively. Moreover, tetrazolium test (TTZ) was applied to check for seed viability. We classified seeds as viable or nonviable after the TTZ test following the method described in Gallo et al. (2015). Note that each year, new individual plants were selected to measure flower traits and conduct reproductive experiments, as some of the previously selected plants were not flowering.

### Flower visitation and florivory by lizards

To identify flower visitors and estimate their visitation frequency (i.e. number of visits and number of visited flowers per time), we conducted one observational census of 15–30 minutes, from 1 to 2 m away from the focal plant, on a total of 56 plant individuals (996 min. in total) from 2019 to 2021 (38 individuals in 2019, 14 in 2020, and 6 in 2021). Animals were considered as potential pollinators whenever they touched the reproductive organs of the flower. For each census, we recorded the start and end time, total number of open flowers in the plant, number of flowers observed during the census, flower visitor species, number of individuals for each flower visitor, and number of flowers visited per flower visitor. When the visitors were lizards, we also noted their age (juvenile or adult), sex (female or male) and floral use (legitimate visit or florivory) to account for intraspecific differences that may affect plant reproduction. Insects that were difficult to identify in the field with photographs alone were captured and taken to the lab. All censuses were conducted between 9:30 am and 6:00 pm on sunny, windless days.

From December 2020 to February 2021, we also quantified florivory rate by lizards in 25 reproductive individuals previously marked in 2018. We recorded the total number of open flowers in the plant, the proportion of flowers with partial florivory (i.e. only petals) and the proportion of flowers with total florivory (i.e. petals plus reproductive organs) over the total number of open flowers per plant. Additionally, to investigate potential morphometric differences depending on lizard sex, a total of 35 adults, 18 females and 17 males, were trapped by hand with noose poles, and marked with a temporary spot of nail polish to avoid resampling the same individual, in May and October 2018. Snout-vent length (SVL), gape width and skull length were measured by using a digital calliper with a 0.01 mm precision. Juvenile lizards were generally observed to be considerably smaller than adult males and females, although we did not directly measure juvenile lengths. Since we measured adult lizards, we were able to easily distinguish juveniles by their visibly smaller size when handled.

### Statistical analyses

To evaluate differences in flower traits between flower morphs (female *vs.* male), we fitted different linear models (LM) for corolla length, corolla diameter at opening, stamen length, and pistil length. Corolla diameter was log-transformed prior to analysis for a better residuals fit. Flower morph was included as the main predictor variable. Corolla length was included as a covariate in the models of stamen length and pistil length to account for flower size differences.

To investigate the reproductive system of *W. frutescens,* we first fitted a generalized linear mixed model (GLMM) with fruit set as the response variable and pollination treatment (open pollination, autogamy, cross hand-pollination) as the main predictor variable. Plant width, plant height, and the mean number of open flowers were excluded from the analyses due to the lack of significance. The response variable was fitted using a binomial error distribution with a logit link function. Second, we built a GLMM with the number of seeds per fruit as response variable (fitted with a Poisson error distribution and a log link function) and two linear mixed models (LMM) with seed weight and seed width as response variables, using pollination treatment as the main predictor variable. Individual plant was always considered as random effect in all models. Finally, we fitted a generalized linear model (GLM) for seed viability (estimated by TTZ test as proportion of TTZ positives) due to singularity problems when adding a random effect. Seed viability (0 *vs* 1) followed a binomial error distribution with a logit link function.

Intraspecific differences in the probability of florivory by the lizard *P. lilfordi* were evaluated by constructing a GLM. The probability of florivory (0 *vs.* 1) per each visiting individual lizard was used as the response variable and the visiting lizard group (juvenile, female or male) as the predictor variable. The response variable was fitted using a binomial error distribution with a logit link function. The total number of open flowers was excluded as a covariate from the analyses, as no effect was detected on the response variables. To assess whether morphometric measurements differ between female and male lizards, we applied t-tests on gape width, SVL, and skull length as response variables and lizard sex (female or male) as the predictor variable. Given the strong positive correlation among these three response variables, we show only the results for gape width, as it likely represents the most critical factor limiting lizard access to the flower. Statistical analyses were not applied to evaluate differences in the visitation frequency between lizards and insects due to the small number of recorded visits by insects.

Models were fitted with the R functions *t.test*, *lm* and *glm* {stats}, and *lmer* and *glmer* {lme4}. The package {DHARMa} was applied for residual diagnostics (R Core Team 2024).

## Results

### Flower traits

**Figure 2.**
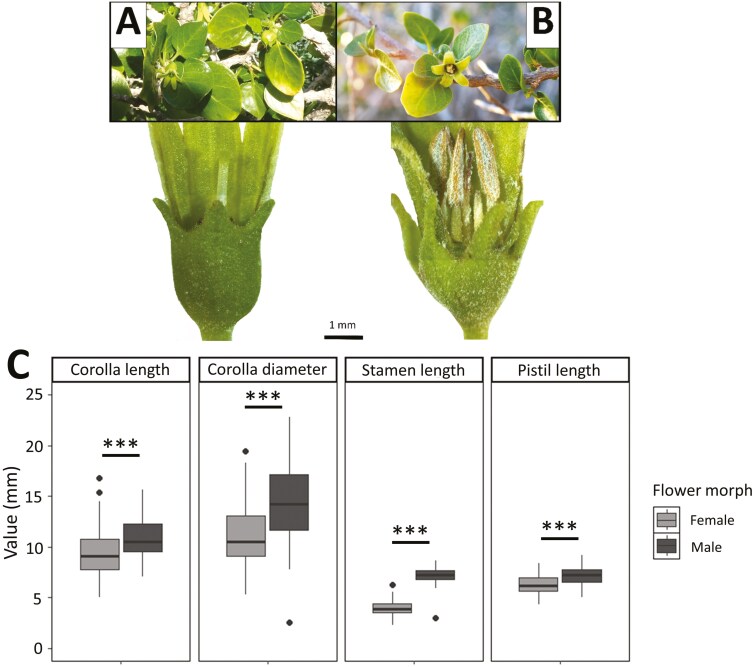
(A) Functional female plant of *W. frutescens*, bearing typical flowers with a longer functional pistil and shorter sterile anthers (brown structure located behind the calyx in the image), as well as fruits. (B) Functional male plant of *W. frutescens,* bearing typical flowers with functional anthers of similar length to the sterile pistil. Bar represents scale (1 mm) of the magnifying glass photographs. (C) Corolla length, corolla diameter, stamen length and pistil length (mm) by flower morph: female (*n* = 146 for all traits, except pistil length where *n* = 145) or male (*n* = 114). Asterisks denote statistical significance: *** *P* < 0.001.

Floral architecture of *W. frutescens* is similar to other solanaceous species: actinomorphic and pentamerous flowers, with a typical bell shape and a pale greenish-yellow colour. On average, flowers showed a corolla length of 10.0 ± 0.1 mm (range = 5.0–16.8, *n* = 260), a corolla diameter at opening of 12.6 ± 0.2 mm (range = 2.5–22.8, *n* = 260), a stamen length of 5.4 ± 0.1 mm (range = 2.3–8.6, *n* = 260), and a pistil length of 6.7 ± 0.1 mm (range = 4.3–9.2, *n* = 259). Although no sexual differences were evident at first glance, two functional flower morphs were revealed when floral traits and reproductive organs (pistil and stamens) were carefully examined. Interestingly, each monitored plant individual (*n* = 19) consistently displayed only one flower morph, which remained unchanged over time (from 2018 to 2021). Typically, female flowers (*n* = 146) showed a longer and functional pistil with shorter and sterile anthers (Fig. 2A), while male flowers (*n* = 114) had functional anthers of similar length to the sterile pistil (Fig. 2B). Male flowers had around 1.2-fold longer corolla length (*F* = 32.32, df = 1, *P <* 0.001) and wider corolla diameter (*F *= 59.78, df = 1, *P <* 0.001) compared to female flowers (Fig. 2C). Moreover, the stamen was 1.7-fold longer in male flower (*F* = 1395.49, df = 1, *P <* 0.001), while pistil length was more similar between flower morphs but statistically longer in males (*F* = 32.78, df = 1, *P <* 0.001; Fig. 2C). Both flower morphs were seen to produce nectar (Authors, *pers. obs*.).

### Fruit set and seed viability

**Figure 3.**
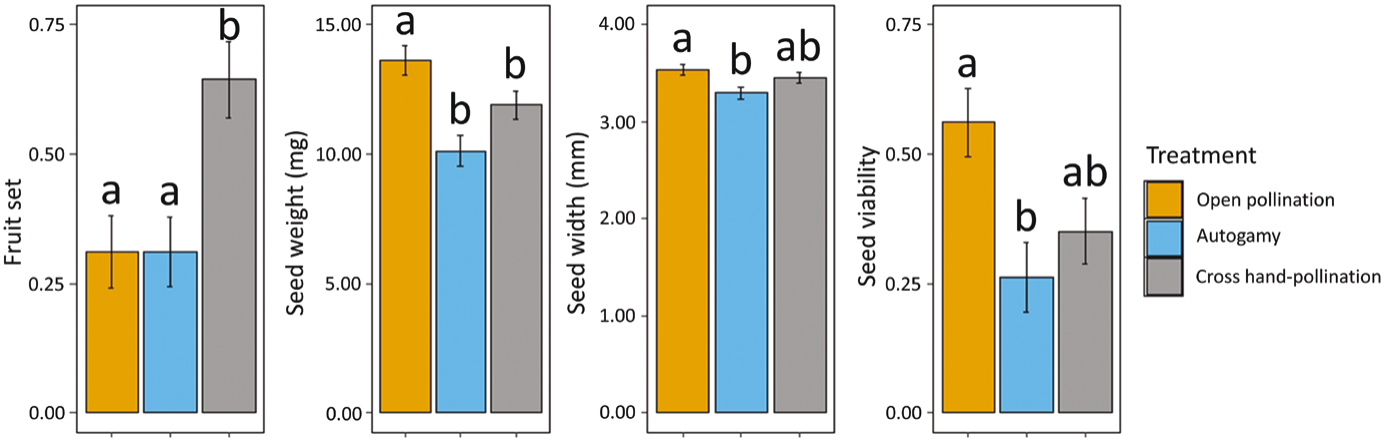
Fruit set (number of fruits/number of bagged flowers; *n* = 10 plants per treatment), seed weight (mg), seed width (mm) and seed viability (proportion of TTZ positives; *n* = 57, 42, and 57 seeds for open pollination, autogamy, and cross hand-pollination treatments, respectively) depending on the pollination treatment for female plants. Note that fruit set for male plants was zero. Bars represent adjusted means ± standard error from post-hoc Tukey test. Different letters denote significant differences between paired levels in post-hoc comparisons.

Functional male flowers (*n* = 364) did not produce any fruit regardless of pollination treatment, whereas 38.01% of the functional female flowers (*n* = 413) resulted in fruit. Fruit set varied among pollination treatments (*χ*^2^ = 31.15, df = 1, *P <* 0.001; Fig. 3), cross hand-pollination yielding more than twice the fruit set compared to the other two treatments (*t* = −4.59 and *P* < 0.001 for open/cross hand-pollination; *t* = −6.06 and *P *< 0.001 for autogamy/cross hand-pollination). By contrast, fruit set was similar between open pollination and autogamy (*t* = 0.008, *P* = 1.00).

Regarding seed viability, a total of 156 seeds (from 37 fruits, 4.24 ± 0.39 seeds per fruit on average) were measured. The number of seeds per fruit was similar among treatments (*χ*^2^ = 3.25, df = 2, *p *= 0.20). However, both seed weight and seed width differed significantly (*χ*^2^ = 25.67, df = 2, *P *< 0.001 and *χ*^2^ = 10.86, df = 2, *P *= 0.004, respectively; Fig. 3). Seeds from open pollination were significantly heavier than those from the autogamy and cross hand-pollination treatments (*t* = 4.61 and *P <* 0.001 for open pollination/autogamy; *t* = 3.24 and *P *= 0.004 for open pollination/cross hand-pollination). Seeds from open pollination were also wider, although the differences between this and the cross hand-pollination treatments were nonsignificant (*t* = 1.63 and *P *= 0.23). Moreover, seed viability was significantly higher under open pollination (*χ*^2^ = 10.12, df = 2, *P *= 0.006), being twice as high as in the autogamy treatment (*z* = 2.91 and *P* = 0.01).

### Flower visitation and florivory

**Figure 4.**
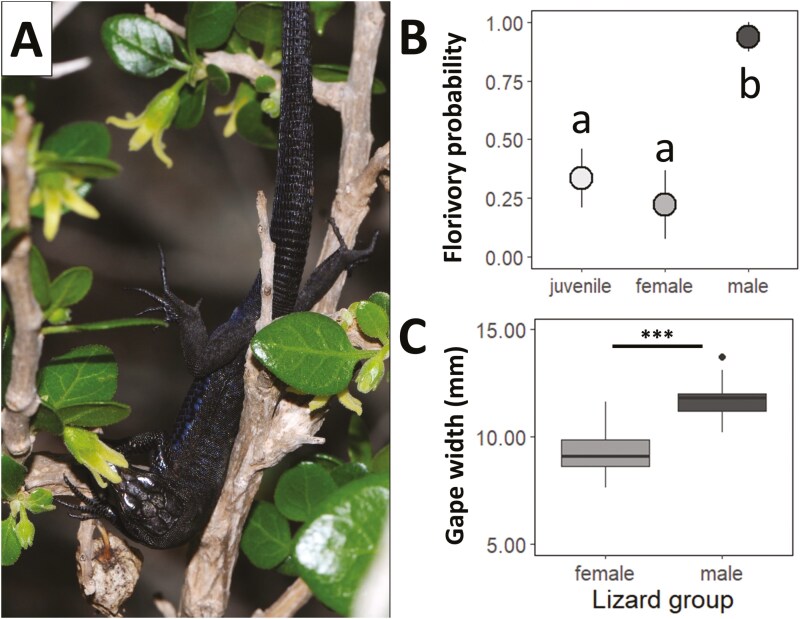
(A) Individual of *P. lilfordi* legitimaly visiting a flower of *Withania frutescens* flower. (B) Mean probability of florivory on *W. frutescens* flowers depending on the lizard group: juveniles (*n* = 15), females (*n* = 9), and males (*n* = 16). Points represent the mean probability, lines the upper and lower standard errors, and different letters denote significant differences between paired levels in post-hoc comparisons. (C) Gape width (mm) of *P. lilfordi* depending on the lizard group (female, *n* = 18; male, *n* = 17). Asterisks denote statistically significant differences: *** *P <* 0.001.

Overall, 44.6% of the plant individuals (*n* = 56), observed between 2019 and 2021, received at least one visit to their flowers. Among the recorded visits (*n* = 54), 75.9% were made by the lizard *P. lilfordi* and 24.1% by insects (specifically, 11.1% by the fly *Helina reversio,* 5.6% by the hoverfly *Meliscaeva auricollis*, 3.7% by thrips, Thysanoptera, and 3.7% by aphids, Aphidoidea). Notably, lizards and insects never visited the same plant concurrently, probably because the latter avoid being preyed upon by the former. Moreover, *P. lilfordi* contacted the highest number of flowers per minute compared to other species (Table 1, Fig. 4A).

**Table 1.** Total number of visits, mean number (± SE) of visits per unit time, and mean number of visited flowers per unit time in *Withania frutescens* during the flowering periods of 2019–2021 on Na Redona (Cabrera archipelago, Balearic Islands).

Order	Family	Visitor species	N total visits	N visits·min^-1^	N flowers·min^-1^
**Lizards**					
Squamata	Lacertidae	*Podarcis lilfordi*	41	0.14 ± 0.02	0.24 ± 0.05
**Insects**					
Diptera	Muscidae	*Helina reversio*	6	0.08 ± 0.01	0.09 ± 0.02
	Syrphidae	*Meliscaeva auricollis*	3	0.07 ± 0.00	0.09 ± 0.02
Thysanoptera	-	-	2	0.13 ± *NA*	0.13 ± *NA*
Homoptera	Aphidoidea	-	2	0.13 ± *NA*	0.13 ± *NA*

Among the 25 individuals of *W. frutescens* monitored in 2020–2021, 72% experienced florivory by lizards (either partial, total or both). Specifically, 72% of the plants showed partial florivory, with a mean intensity of 20 ± 5% of flowers damaged over the total, while 24% experienced total florivory, with a mean intensity of 23 ± 13%. Therefore, *P. lilfordi* was not only the most frequent flower visitor but also a common florivore. In particular, 55% of the lizards observed visiting flowers (*n* = 40; one lizard was excluded from the analysis due to missing data) were found to consume some part of the flowers, either partially or totally. Moreover, there were intraspecific differences in the probability of florivory depending on the lizard age and sex (*χ*^2^ = 18.94, df = 2, *P < *0.001; Fig. 4B). Juvenile and female lizards were less likely to be florivores than male lizards (*z* = −2.9 and *P* = 0.010, from post-hoc comparisons between juveniles and males; *z* = −3.0 and *P* = 0.007, from post-hoc comparisons between females and males), which consumed the flowers in nearly 95% of their visits. Lizard gape width also differed between female and male individuals, being greater in the latter (*t* = −8.08, *P <* 0.001; Fig. 4C).

## Discussion

For the first time, the reproductive biology of *W. frutescens* is described, revealing two flower morphs separated in different individuals, indicating a type of cryptic dioecy. The plant displays a mixed mating system, as both autogamy and open pollination resulted in similar fruit set. However, fruit set was significantly higher under cross hand-pollination, suggesting pollen limitation. Despite this, seed quality and viability were higher under open pollination, highlighting the importance of pollinator visitation in maintaining genetic variability in isolated populations such as that of Na Redona islet. Our study demonstrates the role of the lizard *P. lilfordi* as a legitimate and frequent pollinator of *W. frutescens* which, added to its already known role as seed disperser (Castilla 2000), constitutes a new case of double mutualism on islands (*sensu*Hansen and Müller 2009). We found that flower use varied with lizard age and sex, with juveniles and females mostly acting as legitimate pollinators and males as florivores, likely due to differences in skull length and gape size. This intraspecific variability is consistent with findings in other island plant species (e.g. Fuster and Traveset 2020; Romero-Egea et al. 2023).

### Between self-compatibility and cryptic dioecy

Most flowering plants exhibit hermaphroditic flowers, with individuals acting as both females and males. Only 6–7% of species worldwide have a dioecious system, i.e. with sexes separated in different individuals (Barrett and Hough 2013). Within the Solanaceae family, all dioecious cases described so far are referred to as ‘cryptic dioecy’ or ‘functional dioecy’, when one or both unisexual flower morphs are morphologically hermaphroditic (Li et al. 2010; Penny 2014). One key aspect of this study is the discovery of cryptic dioecy in *W. frutescens*, where two flower morphs—male and female—are separated on different individuals. Male flowers were larger, with functional stamens of the same length as the pistil, whereas female flowers were shorter, with a functional pistil and reduced stamens, very similar to the floral architecture found in the Canarian *W. aristata* by Anderson et al. (2006). The larger size of functionally male flowers is consistent with global trends showing that larger flowers invest more in corollas to enhance pollen export (Paterno et al. 2020). This evolutionary trait could give an advantage to the reproduction of *W. frutescens* in environments with limited pollinator activity as our study system. In contrast to *W. aristata*, though, functional female flowers of *W. frutescens* were able to set fruit by autogamy, displaying a hybrid breeding system between hermaphroditism and dioecy. This has already been described for other *Withania* species, such as *W. ashwagandha* (Mir et al. 2013), in which autogamy resulted in 90.8% fruit set, open pollination in 50.4%, and xenogamy (i.e. cross hand-pollination) in 30.3%. In another *Withania* species, *W. somnifera*, autogamy and open pollination resulted in similar fruit set (~80%), while xenogamy led to a lower fruit set (Kaul et al. 2005). In our study system, cross hand-pollination resulted in a higher fruit set (64.4%) compared to open pollination and autogamy (approx. 31%), suggesting that *W. frutescens* is pollen limited (Knight et al. 2005). However, it is unclear whether this pollen limitation is due to recent or past declines in insect pollinators, or if lizards are inefficient at transferring pollen (Wilcock and Neiland 2002). Although lizards can act as effective pollinators (e.g. Robles et al. 2024) and some studies have reported high pollen adherence on the ventral part of the snout (e.g. Pérez-Mellado et al. 2000), their qualitative contribution might be lower compared to that of insect pollinators. Therefore, pollinator exclusion experiments should be conducted to compare pollination effectiveness between lizards and insects on *W. frutescens* reproduction.

On the other hand, open pollination showed the highest seed quality and viability, suggesting that lizards may enhance genetic diversity and offspring quality of *W. frutescens* by carrying pollen from a wide range of plant individuals around (Wilcock and Neiland 2002; Busch 2005). This must be particularly important for isolated and small plant populations, which are often more vulnerable to genetic bottlenecks and inbreeding (Oostermeijer et al. 2000; Wanderley et al. 2020). Nonetheless, seed size and viability under cross-hand pollination did not differ significantly from either open pollination or autogamy, indicating the need for further research to clarify the extent of lizard-mediated pollen flow. At the same time, the ability of *W. frutescens* to self-pollinate likely ensures reproductive continuity in the event of pollinator failure in these isolated environments (Holsinger 2000; Razanajatovo et al. 2019). This highlights the evolutionary trade-off between reproductive assurance through self-pollination and the genetic benefits of cross-pollination, a common phenomenon in island ecosystems (Crawford et al. 2011).

### Intraspecific variation shifts plant-lizard interaction from mutualism to antagonism

Lizard-flower interactions are common in isolated systems such as islands, where lizard population densities are typically high due to reduced predation and fewer insects. This scarcity of insect pollinators, combined with limited food resources for lizards, may cause a shift in their role from pollinators to florivores (Olesen and Valido 2003; Fuster and Traveset 2020; Justicia-Correcher et al. 2023). Bees are the main flower visitors of other *Withania* species (Kaul et al. 2005; Anderson et al. 2006; Mir et al. 2013). However, in our study, we observed bees—specifically *Amegilla albigena*—visiting flowers only once, and other insects, such as flies and hoverflies, were also rare. Instead, the Balearic lizard *P. lilfordi* was the main flower visitor and potential pollinator of *W. frutescens*, a role recently documented for other plant species on Cabrera Island (Romero-Egea et al. 2023; Robles et al. 2024). Previous studies (Castilla 2000) also confirmed that *P. lilfordi* effectively disperses *W. frutescens* seeds; thus, our research provides a new case of ‘double mutualism’ (Hansen and Müller 2009, Fuster et al. 2019). Similar to the Mediterranean shrub *Cneorum triccocon* (Fuster and Traveset 2020), we expect that juvenile and female lizards act as legitimate pollinators, while male lizards perform better as seed dispersers. This dual role has been commonly observed on islands, suggesting that isolated conditions may have driven the evolution of both flower visitation and seed dispersal in Lacertilia (Fuster et al. 2019; Valido and Olesen 2019; Kahnt et al. 2023).

Nonetheless, *P. lilfordi* also acts as an antagonistic florivore of *W. frutescens*, a behaviour observed in other plant species such as *Medicago arborea, Lavatera maritima* and *Allium subvillosum* on Na Redona islet (Romero-Egea et al. 2023), and other systems (e.g. Gomes et al. 2014; Justicia-Correcher et al. 2023). For instance, Romero-Egea et al. (2023) reported that 65% of *P. lilfordi* visits to plants were legitimate pollination events, while 35% were florivory interactions. In our study, more than half of the lizard visits to *W. frutescens* involved florivory. Interestingly, the probability of florivory was influenced by lizard age and sex, with only 25% of visits by juveniles and females involving florivory, compared to 95% of visits by males. Romero-Egea et al. (2023) also found intraspecific variability in florivory, although the differences between lizard groups were smaller than in our study, probably because they applied a community-level approach. This variability is likely due to the larger size of male *P. lilfordi*, which prevents them from accessing the corolla without damaging the flower. Additionally, larger males may require more energy than flower nectar or scarce insects can provide, leading to the consumption of the entire floral structure (Romero-Egea et al. 2023). Such intraspecific variation can have significant consequences for plant reproduction (González-Varo and Traveset 2016; Zwolak 2018; Fuster and Traveset 2020). Therefore, further research is needed to better understand both mutualistic and antagonistic aspects of plant-lizard interactions and their impact on plant reproductive success.

Comparing the reproductive biology of *W. frutescens* between island and mainland populations in future studies could reveal important shifts driven by environmental constraints, as seen in other plant species. Autogamy, for instance, is often associated with island colonization (Crawford et al. 2011; Grossenbacher et al. 2017). Conversely, different levels of dioecy may evolve to promote outcrossing in response to limited pollinators, as observed in the cryptic dioecious *W. aristata* in the Canary Islands (Anderson et al. 2006). Island ecosystems are also frequently associated with less specialized and less diverse plant-pollinator interactions due to their simplified pollinator communities (Crawford et al. 2011; Traveset et al. 2016). For instance, a study on 54 species of Caribbean Gesneriaceae found that generalized pollination strategies were more common on islands compared to the mainland (Martén‐Rodríguez et al. 2015). In addition, a higher diversity of insect pollinators on the mainland may enhance pollination efficiency, thereby reducing the reliance on self-pollination, in contrast to island populations. While mainland populations benefit from a greater diversity of insect pollinators (Traveset et al. 2016), island populations are also visited by vertebrate pollinators, such as birds and lizards, which play a significant role in pollination (Olesen and Valido 2003). However, as discussed in this study, these vertebrate pollinators can also act as herbivores, potentially affecting plant fitness. As a result, mainland populations of *W. frutescens* are likely to exhibit lower levels of autogamy and dioecy compared to island populations, where more flexible reproductive strategies are required to compensate for pollinator limitations.

## Concluding remarks

Our study describes the first detailed account of the reproductive and pollination systems of the understudied solanaceous *W. frutescens* on a Mediterranean islet, and identifies the Balearic lizard *P. lilfordi* as both the most frequent flower visitor and an important florivore. The mixed mating system of *W. frutescens* suggests a flexible reproductive strategy likely evolved to cope with the unpredictable pollinator availability typical of insular environments. Despite the high florivory rates, lizards appear to play an outsized role in maintaining the reproductive success and genetic variability of insular plant populations. Future research should focus on comparative studies between island and mainland populations to explore how pollination systems evolve in response to ecological constraints. Given the vulnerability of insular plants (Traveset et al. 2016; Gray 2019), understanding their reproductive ecology is critical for informing effective conservation strategies.

## Data Availability

Data and R code are available online at https://figshare.com/s/ea0829be4cf8faab7ba0.
